# Successful treatment of PGAD/GPD with leuprolide: a case report

**DOI:** 10.3389/fpsyt.2025.1623256

**Published:** 2025-08-05

**Authors:** Wang Rong, Hongwen Zhou, Hongqi Fan

**Affiliations:** ^1^ Department of Endocrinology, The First Affiliated Hospital with Nanjing Medical University, Nanjing, China; ^2^ Dpartment of Endocrinology, Lianyungang Affiliated Hospital of Nanjing University of Chinese Medicine, Lianyungang, China

**Keywords:** leuprolide, case report, treatment, PGAD, generalized pareto distribution (GPD)

## Abstract

Persistent genital arousal disorder/genitopelvic dysesthesia (PGAD/GPD) is a rare clinical condition of uncertain etiology. It is characterized by involuntary genital arousal occurring in the absence of sexual interest or desire, and may be accompanied by abnormal sensations in the pelvic and reproductive regions. PGAD/GPD exerts a profound negative impact on patients’ physical and mental health, severely impairing daily functioning and, in some cases, leading to suicidal ideation. This case highlights the potential role of COVID-19 as a triggering factor in the development of PGAD/GPD. The marked improvement in symptoms following treatment with leuprolide suggests that dysregulation of gonadotropin/GnRH signaling may constitute a key pathogenic mechanism underlying this condition. We anticipate that this successful treatment case will provide valuable insights into the etiology and treatment strategies of PGAD/GPD.

## Introduction

Persistent genital arousal disorder/genitopelvic dysesthesia (PGAD/GPD) is a rare condition affecting women’s sexual health, occurring at any age but most commonly after puberty (1). The condition is characterized by sudden and frequent spontaneous genital arousal, often accompanied by genitopelvic sensory abnormalities. Its nature differs from sexual arousal associated with sexual desire or subjective arousal. Symptoms of PGAD/GPD are typically not alleviated through masturbation or orgasm. It is fundamentally distinct from hypersexuality, which is defined by excessive sexual desire that may or may not include persistent genital arousal, whereas PGAD/GPD involves persistent genital arousal in the absence of sexual desire (2). This condition primarily affects women. Due to the unique nature of its symptoms, patients often experience psychological distress, including shame and guilt. Severe cases may be accompanied by anxiety, depression, or even suicidal ideation, significantly impairing quality of life.

However, there is currently limited global understanding of this condition, with only a few reports available. Existing treatment approaches are primarily exploratory in nature. Clinical efficacy remains poor, and follow-up data are scarce. Here, we report a case of PGAD/GPD treated with leuprolide, which demonstrated marked clinical improvement and was followed for nearly two years. The results are summarized below.

## Case report

A 77-year-old female patient presented to our endocrinology outpatient clinic in February 2023 with a chief complaint of “recurrent spontaneous genital arousal for over 50 days.” At the time of presentation, she expressed shame and reluctance to speak, and her medical history was primarily provided by family members. Approximately 50 days after recovering from a COVID-19 infection, she developed spontaneous genital arousal characterized by sudden urges that were not alleviated by intercourse. These symptoms were accompanied by lower limb weakness, pelvic congestion, a burning sensation, and suicidal thoughts. There was no genital swelling or pain. Subsequently, the symptoms recurred intermittently with increasing frequency and duration, eventually becoming persistent without spontaneous resolution. Three days prior to presentation, hormonal tests conducted at another hospital confirmed postmenopausal status, and pelvic MRI revealed no significant abnormalities. Her medical history includes a modified radical mastectomy for right breast invasive ductal carcinoma performed seven years ago at another institution. Postoperatively, she has been maintained on 2.5 mg of letrozole daily. She denies any history of anxiety, depression, or hyperactive sexual desire. Menarche occurred at age 14, and menopause at age 50. She has had four pregnancies and has one son and one daughter. She has reported low spontaneous sexual desire over the past 20 years.

**Figure 1:**
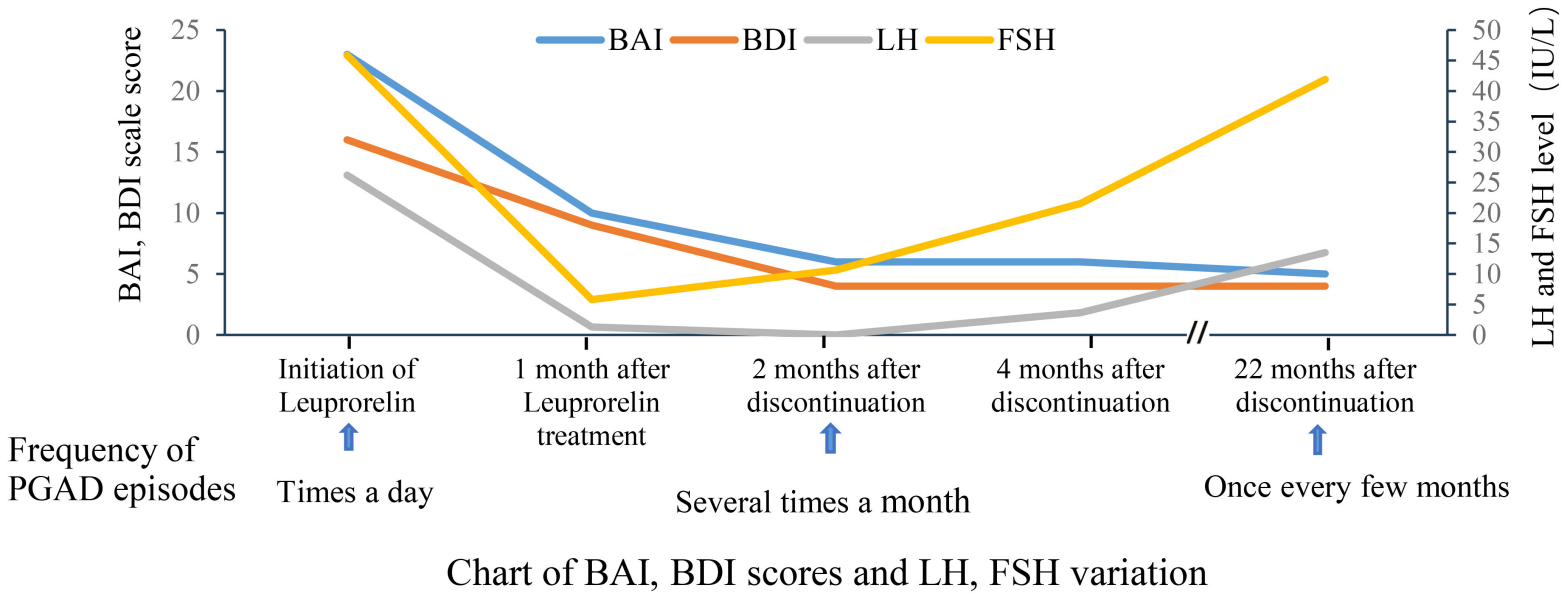
Changes in BAI and BDI Scores and LH Levels.

Based on a comprehensive analysis of the patient’s medical history and clinical presentation, a diagnosis of PGAD/GPD was made. The patient expressed recurrent suicidal ideation and demonstrated a strong desire for treatment. She was informed that no evidence-based treatment guidelines currently exist for PGAD/GPD, and that available therapeutic approaches are largely exploratory. Following approval from the Ethics Committee of the First Affiliated Hospital with Nanjing Medical University and written informed consent, a monthly subcutaneous injection of 3.75 mg leuprolide acetate sustained-release microspheres was initiated. Before treatment and during follow-up, the patient completed assessments using the Beck Anxiety Inventory (BAI) and the Beck Depression Inventory (BDI). Prior to treatment, the BAI score was 23 (15–25 indicates mild anxiety), and the BDI score was 16 (greater than 15 suggests possible depression), indicating mild anxiety and possible depression (**Table 1**, **Figure 1**).

**Table 1:** Relevant laboratory examinations and BAI and BDI scores before, during and after treatment.

	Initiation of Leuprorelin	1 month after Leuprorelin treatment	2 months after discontinuation	4 months after discontinuation	22 months after discontinuation
date of hospital visiting	2013.02.17	2023.03.25	2023.07.07	2023.09.08	2025.03.21
LH(IU/L)	26.2	1.27	<0.01	3.64	15.87
FSH(IU/L)	45.86	5.8	10.62	21.58	41.88
E2(pmol/L)	24.33	<55.07	<55.07	<55.07	<55.07
T(nmol/L)	<0.1	<0.01	0.1	0.18	0.46
P(nmol/L)	0.6	0.71	1.27	0.72	0.77
PRL(mIU/L)	848.8	232.36	189.25	134.51	148.02
DHEAS(umol/L)	–	1.3	1.3	1.2	1.6
BAI score	23	10	6	6	6
BDI score	16	9	4	4	4

LH, Luteinizing Hormone; FSH, Follicle-stimulating Hormone; E2, Estradiol; T, Testosterone; P, Progesterone; PRL, Prolactin; DHEAS, dehydroepiandrosterone; BAI, Beck Anxiety Inventory; BDI, Beck Depression Inventory.

The patient reported a worsening of genital arousal symptoms within the first three days following leuprolide administration, which gradually improved after one week. One month later, symptoms showed marked improvement, with the BAI score reduced to 10 and the BDI score reduced to 9. Follow-up hormone levels were consistent with the physiological changes expected after Gonadotropin-Releasing Hormone Analog (GnRHa) therapy. Two months after treatment, the symptoms were largely absent, with only occasional brief recurrences that resolved spontaneously. At the three-month follow-up, the patient reported complete remission of symptoms, and leuprolide therapy was discontinued, with BAI and BDI scores of 5. Over the three-month treatment period, three injections of leuprolide acetate sustained-release microspheres were given.

One month after discontinuing leuprolide, approximately 10 days following reinfection with COVID-19, a mild recurrence of sexual arousal symptoms was noted, though milder than previous episodes. The main symptom was a burning sensation in the perineal region, which typically occurred during leisure time and resolved with cognitive distraction. Two months after discontinuation, the patient returned for a follow-up visit and reported that symptoms had largely resolved, with occasional recurrence triggered by watching videos on a smartphone, which resolved with focused distraction. Re-evaluation of sex hormones showed that LH and FSH levels remained below postmenopausal baseline. The BAI score was 6, and the BDI score was 4. Four months after medication discontinuation, the patient reported two episodes over the preceding two months, both of which resolved quickly with mild intensity. Follow-up hormone levels indicated a gradual return of FSH and LH to postmenopausal levels. The BAI and BDI scores remained stable at 6 and 4 (**Table 1**, **Figure 1**), respectively. After seven months without medication, the patient experienced a resurgence of symptoms following attendance at a wedding banquet. These episodes lasted approximately 30 minutes and were accompanied by lower limb weakness and mild pelvic discomfort. Symptoms recurred intermittently every 2–3 days over a three-month period before resolving spontaneously. At the 22-month telephone follow-up, the patient reported mild and transient episodes of spontaneous genital arousal, which resolved without intervention. Hormone reassessment confirmed postmenopausal status with LH levels persistently lower than baseline. The BAI and BDI scores were 6 and 4, respectively.

## Discussion

In 2001, Leiblum and Nathan first reported five cases of Persistent Sexual Arousal Syndrome (PSAS) in women, characterized by symptoms of unrelieved genital arousal (3). Considering that the condition was primarily genital in nature rather than sexual, Leiblum renamed it Persistent Genital Arousal Disorder (PGAD) in 2006 (4). In 2019, the International Society for the Study of Women’s Sexual Health (ISSWSH) convened a panel of experts to re-evaluate the terminology. The panel recognized that PGAD did not fully capture the range of symptoms experienced by patients with genitopelvic dysesthesia (GPD). As a result, the term PGAD was broadened to encompass GPD. The term PGAD/GPD maintains a primary focus on persistent arousal symptoms while acknowledging the associated physical manifestations (1).

Currently, the epidemiological characteristics and pathophysiological mechanisms of PGAD/GPD are not well understood. Due to its unique symptoms and privacy concerns, many patients experience self-shame or even guilt, which often leads to avoidance of medical consultation or delayed treatment. Additionally, clinicians frequently lack sufficient understanding of the condition, further contributing to the unclear epidemiological characteristics of this disorder to date. Epidemiological studies conducted abroad have reported prevalence rates ranging from 0.6% to approximately 3% (5, 6), suggesting that a considerable number of individuals may be affected globally. Limited information exists on the pathophysiology of PGAD/GPD, partly due to the absence of validated animal models for experimental study. Although a few international case reports have proposed various etiological hypotheses in recent years, these are generally unsupported by robust clinical or laboratory evidence due to the small number of cases. Current hypotheses include vascular changes, central or peripheral nerve dysfunction, Tarlov cysts, psychological, pharmacological, dietary, and hormonal factors, neurotransmitter imbalances, mechanical pressure on genital structures, or a combination of these (7–9). Given the limited understanding of disease mechanisms, clinical management remains largely exploratory, focusing on presumed etiologies. Systematic evaluations of treatment efficacy and safety through clinical trials are lacking. Most therapeutic approaches are drawn from case reports and may involve trigger removal, local anesthetics, cognitive-behavioral therapy, mindfulness techniques, hypnotherapy, pelvic floor physical therapy, electroconvulsive therapy, pharmacological therapies (such as antidepressants, anxiolytics, GnRHa, among others), or surgical intervention in cases involving Tarlov cysts (10–12). Reported outcomes for these treatments vary significantly. Deka et al. reported the successful treatment of a case of PGAD/GPD using leuprolide, but the treatment lacked psychological assessment and follow-up data after discontinuation of treatment (13). Our case report further supports the potential efficacy of GnRHa in PGAD/GPD management and contributes detailed symptom tracking and follow-up information across the treatment continuum.

The patient exhibited marked spontaneous genital arousal symptoms following two laboratory-confirmed COVID-19 infections. The underlying mechanism is hypothesized to involve COVID-19-mediated activation of the hypothalamic-pituitary-gonadal (HPG) axis, leading to increased pulsatile secretion of gonadotropin-releasing hormone (GnRH) and subsequent symptom development and exacerbation of PGAD/GPD. During the initial phase of leuprolide treatment (within the first three days post-injection), the patient’s symptoms worsened, which may be attributed to the “flare-up effect” induced by GnRH agonists. Subsequently, symptoms rapidly improved, likely due to suppression of the HPG axis. Based on laboratory test results and clinical progression, we speculate that leuprolide may suppress pulsatile GnRH secretion, thereby inhibiting pituitary luteinizing hormone (LH) levels and improving the patient’s symptomatology. Following the discontinuation of leuprolide acetate, both LH and FSH levels gradually returned to pre-treatment values; however, the patient’s symptoms did not worsen significantly. Potential underlying mechanisms include the following: (1) GnRHa may induce a “resetting” effect on the HPG axis, thereby suppressing hormonal signaling pathways associated with PGAD/GPD (14); (2) GnRHa may modulate relevant neuroendocrine pathways through epigenetic mechanisms (15). Furthermore, 22 months after treatment cessation, FSH returned to pre-treatment levels, while LH remained relatively suppressed. This may be attributable to the more potent and sustained inhibitory effect of GnRHa on the LH secretion pathway (16). The dissociation between LH suppression and the recovery of FSH to pre-treatment levels may indicate a stronger correlation between LH dynamics and clinical manifestations. Notably, a significant positive correlation between LH and sexual arousal has been well-documented, as demonstrated by studies on male and female pre-ovulatory libido peaks (17, 18). Additionally, BAI and BDI scores showed significant improvement (**Figure 1**, **Table 1**), suggesting that anxiety and depressive symptoms may be secondary to the underlying physical condition. This may partially account for the limited efficacy of current international treatment regimens that rely solely on psychotropic medications for managing PGAD/GPD.

Notably, the incidence of central precocious puberty among adolescents has significantly increased during the COVID-19 pandemic. For example, in a cohort study of school-aged girls in Shanghai, the incidence of precocious puberty in 2020 was significantly higher than in 2016–2019, accompanied by elevated GnRH levels (19). GnRH neurons originate from the olfactory bulb, develop in the olfactory cortex, and eventually mature in the hypothalamus (20). COVID-19 infection is frequently associated with olfactory dysfunction, and live viruses have been isolated from hypothalamic tissue, supporting the hypothesis of direct viral effects on hypothalamic regulation (21). To date, no studies have specifically investigated the impact of COVID-19 on LH levels in postmenopausal women. A recent meta-analysis reported that, compared to the control group, COVID-19 patients exhibited significantly elevated LH levels, a marked reduction in the FSH/LH ratio, and no significant changes in estradiol concentrations (22). Additionally, post-COVID-19 infection has been linked to increased expression of Kiss1 and its receptor GPR54, which is believed to enhance GnRH pulse frequency (23). GnRH and its receptors are not only localized in the hypothalamus but are also widely distributed throughout the limbic system and cerebral cortex, particularly the prefrontal cortex, where they play roles in regulating sexual desire and impulse control via modulation of the dopaminergic (DA) pathway (24, 25). Although the mechanism by which COVID-19 infection triggers PGAD/GPD by interfering with the HPGA axis remains unclear, COVID-19 infection and its associated physiological and psychological changes may disrupt the GnRH pulse rhythm and amplitude, potentially contributing to its onset.

## Conclusions

In conclusion, PGAD/GPD is a complex and under-recognized disorder with a potentially multifactorial etiology. Treatment should be individualized to target the most likely underlying cause, as hormone-suppressive therapy may not be suitable for all patients. The present report describes a unique clinical scenario (a surgical history of breast cancer), thereby providing a formal indication for GnRHa use. However, given that the application of GnRHa for PGAD/GPD is off-label and the lack of randomized controlled trial data supporting its application, the generalizability of this approach remains limited. Nevertheless, it offers novel insights into the pathophysiology of the disease and potential strategies for clinical management. Given women’s relatively conservative attitudes toward “sex,” as exemplified by the patient in this case who was hesitant to speak during her initial outpatient visit, feelings of shame and embarrassment may prevent patients from actively seeking medical help. In addition, clinical physicians generally lack sufficient understanding of PGAD/GPD. Through this case, we aim to raise awareness among both patients and clinicians—including but not limited to endocrinologists, urologists, gynecologists, psychiatrists, and neurologists—to improve recognition and treatment of PGAD/GPD in future clinical practice.

## Data Availability

The original contributions presented in the study are included in the article/supplementary material. Further inquiries can be directed to the corresponding author.
